# Secretion of early and late substrates of the type III secretion system from *Xanthomonas* is controlled by HpaC and the C-terminal domain of HrcU

**DOI:** 10.1111/j.1365-2958.2010.07461.x

**Published:** 2011-01

**Authors:** Christian Lorenz, Daniela Büttner

**Affiliations:** Institute of Biology, Department of Genetics, Martin-Luther University Halle-WittenbergD-06099 Halle (Saale), Germany

## Abstract

The plant pathogenic bacterium *Xanthomonas campestris* pv. *vesicatoria* utilizes a type III secretion (T3S) system to inject effector proteins into eukaryotic cells. T3S substrate specificity is controlled by HpaC, which promotes secretion of translocon and effector proteins but prevents efficient secretion of the early substrate HrpB2. HpaC and HrpB2 interact with the C-terminal domain (HrcU_C_) of the FlhB/YscU homologue HrcU. Here, we provide experimental evidence that HrcU is proteolytically cleaved at the conserved NPTH motif, which is required for binding of both HpaC and HrpB2 to HrcU_C_. The results of mutant studies showed that cleavage of HrcU contributes to pathogenicity and secretion of late substrates but is dispensable for secretion of HrpB2, which is presumably secreted prior to HrcU cleavage. The introduction of a point mutation (Y318D) into HrcU_C_ activated secretion of late substrates in the absence of HpaC and suppressed the *hpaC* mutant phenotype. However, secretion of HrpB2 was unaffected by HrcU_Y318D_, suggesting that the export of early and late substrates is controlled by independent mechanisms that can be uncoupled. As HrcU_Y318D_ did not interact with HrpB2 and HpaC, we propose that the substrate specificity switch leads to the release of HrcU_C_-bound HrpB2 and HpaC.

## Introduction

Gram-negative plant and animal pathogenic bacteria often employ type III secretion (T3S) systems to deliver bacterial effector proteins directly into eukaryotic cells, a process referred to as translocation (Ghosh, 2004). Type III effector proteins manipulate host cellular pathways to the benefit of the bacteria and thus allow successful multiplication of the bacteria in the host tissue (Block *et al*., 2008; Galan, 2009). Translocation-associated T3S systems are evolutionarily related to flagellar T3S systems, which are the key bacterial motility organelles (Desvaux *et al*., 2006). Both T3S systems consist of a membrane-spanning secretion apparatus (basal body) but differ in their extracellular appendages. The flagellar basal body is linked via an extracellular hook to the flagellar filament, whereas the translocation-associated basal body is connected to a pilus (plant pathogens) or needle (animal pathogens) that serve as protein transport devices to the host–pathogen interface (Ghosh, 2004; Macnab, 2004). The T3S pilus from plant pathogenic bacteria is considerably longer (up to 2 µm) than the T3S needle (40–80 nm) and presumably spans the plant cell wall (Jin and He, 2001; Koebnik, 2001; Li *et al*., 2002; Ghosh, 2004). Needle and pilus are directly or indirectly connected to the bacterial channel-like T3S translocon in the host plasma membrane, which mediates effector protein translocation (Büttner and Bonas, 2002a; Coombes and Finlay, 2005; Mueller *et al*., 2008).

Translocation-associated T3S systems from plant and animal pathogenic bacteria secrete at least three different sets of substrates, i.e. (i) proteins involved in the assembly of the extracellular needle or pilus, (ii) components of the T3S translocon and (iii) effector proteins. Efficient secretion and/or translocation of T3S substrates depends on a signal that is often located in the N-terminal protein region and is not conserved on the amino acid level (Anderson and Schneewind, 1997; Lloyd *et al*., 2001; Petnicki-Ocwieja *et al*., 2002; Arnold *et al*., 2009; Samudrala *et al*., 2009). Furthermore, in some cases bacterial cytoplasmic T3S chaperones are involved that bind to secreted substrates and promote their stability and/or secretion (Parsot *et al*., 2003; Ghosh, 2004).

It is postulated that the secretion of extracellular components of the secretion apparatus precedes effector protein translocation. This implies that the substrate specificity of the T3S system switches from ‘early’ to ‘late’ substrates. In animal pathogenic bacteria the T3S substrate specificity is controlled by so-called T3S substrate specificity switch (T3S4) proteins that are themselves secreted. Examples are YscP from *Yersinia* spp. that switches the T3S substrate specificity from needle to translocon and effector proteins, and FliK from flagellar T3S systems that promotes secretion of filament proteins after hook assembly (Minamino *et al*., 1999a,b; Journet *et al*., 2003; Agrain *et al*., 2005; Sorg *et al*., 2007). The T3S substrate specificity switch depends on the interactions between T3S4 proteins and the cytoplasmic domains of conserved inner membrane proteins that belong to the FlhB/YscU family (Minamino and Macnab, 2000a; Ferris and Minamino, 2006; Waters *et al*., 2007; Botteaux *et al*., 2008). Members of this family contain four transmembrane helices and a C-terminal cytoplasmic domain that is proteolytically cleaved off but probably associates with the remaining part of the protein and was proposed to act as a substrate acceptor site (Allaoui *et al*., 1994; Minamino and MacNab, 2000a,b; Fraser *et al*., 2003; Deane *et al*., 2008; Berger *et al*., 2010). Cleavage of FlhB/YscU family members occurs autocatalytically between the asparagine and proline residues of a conserved NPTH (letters refer to amino acids) motif and results in a reorientation of the PTH loop (Minamino and MacNab, 2000a; Lavander *et al*., 2002; Ferris *et al*., 2005; Sorg *et al*., 2007; Deane *et al*., 2008; Zarivach *et al*., 2008; Björnfot *et al*., 2009; Lountos *et al*., 2009; Wiesand *et al*., 2009). It was proposed that the cleavage and presumably a conformational change in the C-terminal domain of FlhB/YscU family members that is induced upon binding of T3S4 proteins contribute to the T3S substrate specificity switch (Williams *et al*., 1996; Edqvist *et al*., 2003; Ferris *et al*., 2005; Cornelis *et al*., 2006; Deane *et al*., 2008; Minamino *et al*., 2008; Zarivach *et al*., 2008; Björnfot *et al*., 2009; Lountos *et al*., 2009; Wiesand *et al*., 2009). This model is corroborated by the finding that the wild-type phenotype in T3S4 mutants from *Salmonella typhimurium*, *Yersinia pseudotuberculosis* and enteropathogenic *Escherichia coli* can be restored by extragenic suppressor mutations in the C-terminal regions of FlhB, YscU and the homologous EscU protein respectively (Kutsukake *et al*., 1994; Williams *et al*., 1996; Edqvist *et al*., 2003; Zarivach *et al*., 2008).

While the molecular mechanisms underlying control of T3S substrate specificity have intensively been studied in animal pathogenic bacteria, little is known about the mechanisms in plant pathogens. In our laboratory, we study *Xanthomonas campestris* pv. *vesicatoria*, which is the causal agent of bacterial spot disease in pepper and tomato plants and one of the model systems for the analysis of T3S. The T3S system from *X. campestris* pv. *vesicatoria* is encoded by the chromosomal *hrp* (hypersensitive response and pathogenicity) gene cluster, which contains 25 genes that are organized in eight transcriptional units (Bonas *et al*., 1991; Büttner *et al*., 2007; Weber *et al*., 2007). Comparative sequence analysis of *hrp* gene products revealed that eleven proteins (referred to as Hrc for Hrp conserved) are conserved among plant and/or animal pathogenic bacteria (Büttner and Bonas, 2002b; He *et al*., 2004). They probably constitute the core components of the membrane-spanning secretion apparatus. Mutant studies revealed that *hrc* and most *hrp* genes are essential for pathogenicity (Fenselau *et al*., 1992; Fenselau and Bonas, 1995; Wengelnik *et al*., 1996; Huguet and Bonas, 1997; Rossier *et al*., 2000). Only in a few cases, mutations of individual genes of the *hrp* gene cluster do not completely abolish the bacteria–plant interaction. The corresponding gene products were therefore designated Hpa (Hrp associated) and proposed to be involved in the control of T3S (Huguet *et al*., 1998; Büttner *et al*., 2004; 2006; Lorenz *et al*., 2008a,b;). We have previously shown that the efficient secretion and translocation of effector proteins such as AvrBs1, AvrBs3, AvrBsT, XopC, XopJ and XopF1 depend on the T3S chaperone HpaB, which interacts with effector proteins and presumably targets them to the T3S system-associated ATPase HrcN (Büttner *et al*., 2004; 2006; Lorenz *et al*., 2008b). HpaB binds to HpaC, an additional cytoplasmic control protein that promotes secretion of translocon and effector proteins but prevents efficient secretion of HrpB2, which is required for pilus assembly and is therefore presumably one of the first substrates that travels the secretion apparatus (Rossier *et al*., 2000; Weber *et al*., 2005; Lorenz *et al*., 2008b). As HpaC differentially regulates the secretion of early (HrpB2) and late (effector and translocon proteins) T3S substrates, it likely acts a cytoplasmic T3S4 protein. This hypothesis is corroborated by the finding that HpaC interacts with the C-terminal domain of HrcU, which is a member of the FlhB/YscU family of inner membrane proteins (Lorenz *et al*., 2008b). Interestingly, however, HpaC does not interact with the full-length HrcU protein, suggesting that the interaction with the C-terminal domain of HrcU depends on a certain protein conformation that is altered in the context of the full-length HrcU protein (Lorenz *et al*., 2008b). In addition to HpaC, the C-terminal domain of HrcU was shown to interact with HrpB2 but not with other T3S substrates and is therefore presumably not a general T3S substrate acceptor site (Lorenz *et al*., 2008b).

In this study, we investigated the contribution of the T3S4 protein HpaC and the C-terminal cytoplasmic domain of HrcU (HrcU_C_) to T3S of early and late substrates from *X. campestris* pv. *vesicatoria*. The analysis of HrcU derivatives mutated in the NPTH motif suggests that the efficient cleavage of HrcU but not the cleavage event *per se* is required for pathogenicity and T3S of late substrates whereas HrpB2 is presumably secreted prior to HrcU cleavage. The results of protein–protein interaction studies revealed that the NPTH motif of HrcU is required for binding of both HrpB2 and HpaC to HrcU_C_. Notably, the introduction of a P265G mutation into HrcU abolished the HrcU_C_–HrpB2 interaction and also the efficient secretion of HrpB2. In contrast, secretion of HrpB2 was unaffected upon introduction of a point mutation (Y318D) into HrcU_C_, which suppressed the *hpaC* mutant phenotype with respect to pathogenicity and T3S of translocon and effector proteins. We therefore assume that the control mechanisms underlying secretion of early and late substrates can be uncoupled. Given the finding that HrcU_Y318D_ did not interact with HrpB2 and HpaC, the substrate specificity switch in *X. campestris* pv. *vesicatoria* likely leads to the release of HrcU_C_-bound HrpB2 and HpaC.

## Results

### Efficient proteolytic cleavage of HrcU depends on the conserved NPTH amino acid motif

**Fig. 1:**
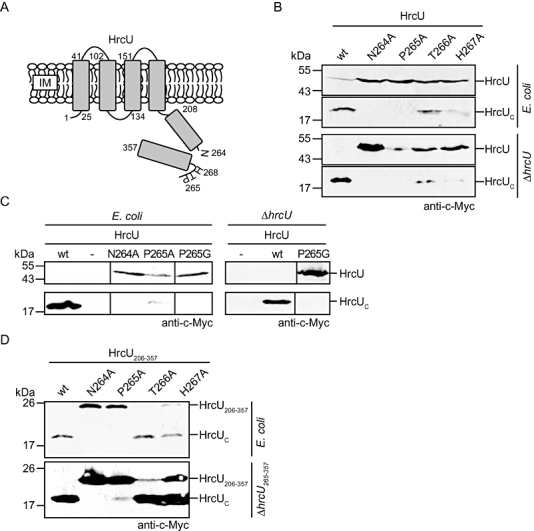
Proteolytic cleavage of HrcU depends on the NPTH motif. A. Schematic representation of HrcU. HrcU contains four transmembrane helices and a C-terminal cytoplasmic region that is proteolytically cleaved. Cleavage presumably occurs at the NPTH motif and results in a conformational change of the PTH loop as was shown for HrcU homologues from animal pathogenic bacteria. Numbers refer to amino acid positions. IM, inner membrane. B. Proteolytic cleavage of HrcU and point mutant derivatives. Equal amounts of total-cell extracts from *E. coli* and *X. campestris* pv. *vesicatoria* strain 85-10Δ*hrcU* (Δ*hrcU*) encoding HrcU-c-Myc (wt), HrcU_N264A_-c-Myc (N264A), HrcU_P265A_-c-Myc (P265A), HrcU_T266A_-c-Myc (T266A) and HrcU_H267A_-c-Myc (H267A), respectively, from corresponding expression constructs were analysed by immunoblotting using a c-Myc epitope-specific antibody. C. HrcU_P265A_-c-Myc is partially cleaved. Equal amounts of total-cell extracts from *E. coli* and *X. campestris* pv. *vesicatoria* strain 85-10Δ*hrcU* (Δ*hrcU*) carrying the empty vector (−) or encoding HrcU-c-Myc (wt), HrcU_N264A_-c-Myc (N264A), HrcU_P265A_-c-Myc (P265A) and HrcU_P265G_-c-Myc (P265G), respectively, from corresponding expression constructs were analysed as described in (B). For the better visualization of the HrcU cleavage product, the blot was overexposed. D. Mutations in the NPTH motif of HrcU_206–357_-c-Myc affect proteolytic cleavage. Equal amounts of total-cell extracts from *E. coli* and *X. campestris* pv. *vesicatoria* strain 85-10Δ*hrcU_265–357_* (Δ*hrcU_265–357_*) encoding HrcU_206–357_-c-Myc (wt), HrcU_206–357/N264A_-c-Myc (N264A), HrcU_206–357/P265A_-c-Myc (P265A), HrcU_206–357/T266A_-c-Myc (T266A) and HrcU_206–357/H267A_-c-Myc (H267A), respectively, from corresponding expression constructs were analysed as described in (B).

The FlhB/YscU homologue HrcU from *X. campestris* pv. *vesicatoria* strain 85-10 contains four transmembrane helices and a C-terminal cytoplasmic region that is proteolytically cleaved in both *E. coli* and *X. campestris* pv. *vesicatoria* (Fig. 1A; Lorenz *et al*., 2008b; Berger *et al*., 2010). Cleavage of HrcU presumably occurs at the conserved NPTH motif (amino acids 264–267) as was described for HrcU homologues from animal pathogenic bacteria. To study the contribution of the NPTH motif of HrcU to protein cleavage and function, we introduced point mutations that led to an exchange of each amino acid residue of the NPTH motif by alanine respectively. The resulting HrcU mutant derivatives were analysed as C-terminally c-Myc epitope-tagged proteins in *E. coli* and *X. campestris* pv. *vesicatoria* strain 85-10Δ*hrcU* by immunoblotting. Using a c-Myc epitope-specific antibody, we detected the full-length HrcU-c-Myc, HrcU_T266A_-c-Myc and HrcU_H267A_-c-Myc proteins and/or corresponding cleavage products (Fig. 1B). As full-length HrcU-c-Myc was only detectable in *E. coli* but not in *X. campestris* pv. *vesicatoria*, we assume that the proteolytic cleavage of HrcU-c-Myc in *X. campestris* pv. *vesicatoria* was nearly complete (Fig. 1B). We detected increased levels of uncleaved HrcU_T266A_-c-Myc and HrcU_H267A_-c-Myc when compared with HrcU-c-Myc, suggesting that mutations of amino acids T266 and H267 of HrcU affect the efficiency of the proteolytic cleavage. The C-terminal HrcU cleavage product was not observed for HrcU_N264A_-c-Myc and only in significantly reduced amounts for HrcU_P265A_-c-Myc (upon overexposure of the blot; Fig. 1B and C). We also introduced an additional mutation into HrcU that led to an exchange of the proline residue at position 265 by a glycine. Notably, the P265G exchange resulted in a complete loss of detectable HrcU cleavage (Fig. 1C).

In addition to the full-length proteins, we generated HrcU derivatives lacking the N-terminal 205 amino acids (HrcU_206–357_-c-Myc). HrcU_206–357_-c-Myc was expressed at higher levels than HrcU-c-Myc, which facilitated the detection of the C-terminal cleavage product. Immunoblot analysis revealed the presence of cleavage products for HrcU_206–357_-c-Myc and corresponding T266A and H267A mutants in both *E. coli* and *X. campestris* pv. *vesicatoria* (Fig. 1D). Cleavage was not observed for HrcU_206–357/N264A_-c-Myc; however, small amounts of the cleavage product were detectable for HrcU_206–357/P265A_-c-Myc, which supports the above finding that proteolytic cleavage is not completely abolished by the P265A mutation (Fig. 1D).

### Mutations in the NPTH motif of HrcU interfere with protein function

To analyse whether HrcU mutant derivatives complement the *hrcU* mutant phenotype, *X. campestris* pv. *vesicatoria* strains 85-10 and 85-10Δ*hrcU* carrying the empty vector or *hrcU* expression constructs were inoculated into leaves of susceptible Early Cal Wonder (ECW) and resistant ECW-10R pepper plants. ECW-10R plants carry the *Bs1* resistance (*R*) gene and induce the hypersensitive response (HR) upon recognition of the type III effector AvrBs1 that is delivered by strain 85-10 (Ronald and Staskawicz, 1988; Escolar *et al*., 2001). The HR is a rapid local plant cell death at the infection site that is activated by a plant *R* gene upon recognition of an individual type III effector [also termed avirulence (Avr) protein; Jones and Dangl, 2006].

**Fig. 2:**
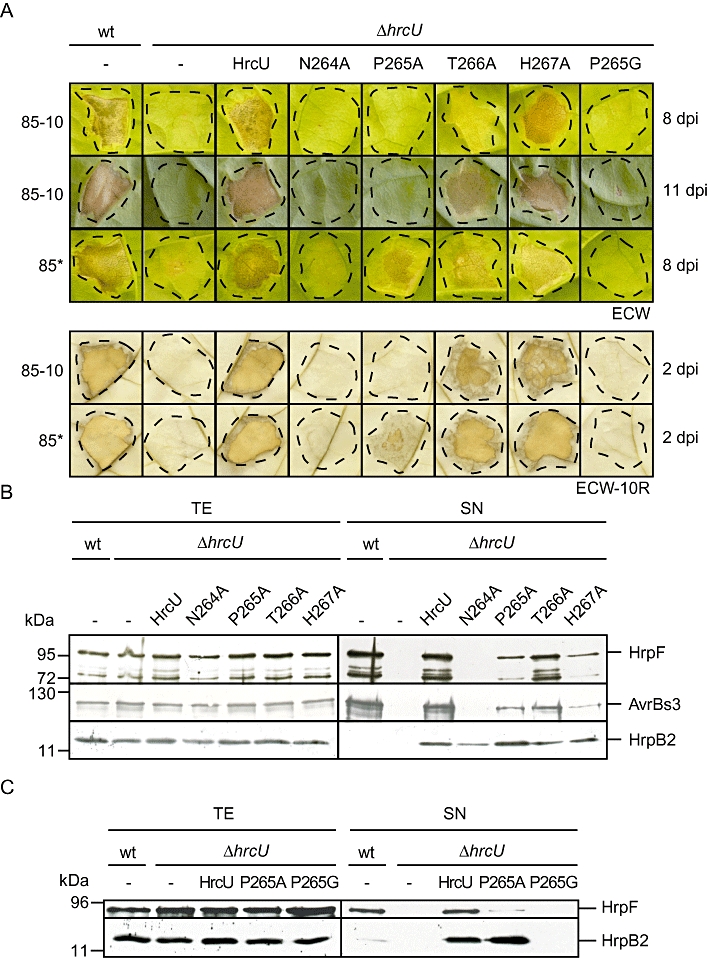
Complementation studies with HrcU mutant derivatives. A. The conserved asparagine residue of the NPTH motif of HrcU is essential for pathogenicity. *X. campestris* pv. *vesicatoria* strains 85-10 (wt), 85* (wt), 85-10Δ*hrcU* (*ΔhrcU*) and 85*Δ*hrcU* (Δ*hrcU*) carrying the empty vector (−) or encoding HrcU-c-Myc (wt), HrcU_N264A_-c-Myc (N264A), HrcU_P265A_-c-Myc (P265A), HrcU_T266A_-c-Myc (T266A), HrcU_H267A_-c-Myc (H267A) and HrcU_P265G_-c-Myc (P265G), respectively, from corresponding expression constructs were inoculated into leaves of susceptible ECW and resistant ECW-10R pepper plants. Disease symptoms were photographed 8 and 11 dpi as indicated. For the better visualization of the HR, leaves were bleached in ethanol 2 dpi. Dashed lines mark the infiltrated areas. B. The N264A mutation abolishes T3S of translocon and effector proteins but does not affect secretion of the pilus assembly protein HrpB2. *X. campestris* pv. *vesicatoria* strains 85* (wt) and 85*Δ*hrcU* (Δ*hrcU*) carrying the empty vector (−) or encoding HrcU-c-Myc (wt), HrcU_N264A_-c-Myc (N264A), HrcU_P265A_-c-Myc (P265A), HrcU_T266A_-c-Myc (T266A) and HrcU_H267A_-c-Myc (H267A), respectively, were incubated in secretion medium. Total-cell extracts (TE) and culture supernatants (SN) were analysed by immunoblotting using antibodies specific for the translocon protein HrpF, the effector protein AvrBs3 (ectopically expressed from construct pDSF300) and HrpB2. C. HrcU_P265G_ does not promote secretion of HrpB2. *X. campestris* pv. *vesicatoria* strains 85* (wt) and 85*Δ*hrcU* (Δ*hrcU*) carrying the empty vector (−), HrcU-c-Myc (HrcU), HrcU_P265A_-c-Myc (P265A) and HrcU_P265G_-c-Myc (P265G), respectively, were incubated in secretion medium. TE and SN were analysed by immunoblotting using HrpF- and HrpB2-specific antibodies respectively.

As expected, strain 85-10 induced water-soaked lesions in ECW and the HR in ECW-10R plants whereas no plant reactions were observed after inoculation of strain 85-10Δ*hrcU* (Fig. 2A). The *hrcU* mutant phenotype was complemented by construct pBRMhrcU, which encodes a C-terminally c-Myc epitope-tagged HrcU derivative under control of the *lac* promoter (Fig. 2A). Partial complementation was observed for HrcU_T266A_-c-Myc and HrcU_H267A_-c-Myc, whereas strain 85-10Δ*hrcU* carrying HrcU_N264A_-c-Myc, HrcU_P265A_-c-Myc and HrcU_P265G_-c-Myc, respectively, did not cause visible plant reactions (Fig. 2A). We also performed infection assays with hrpG* strains that carry a mutated version of the key regulator HrpG and thus constitutively express the T3S genes (Rossier *et al*., 1999; Wengelnik *et al*., 1999). Notably, we observed a partial complementation of the *hrcU* mutant phenotype by HrcU_P265A_-c-Myc but not by HrcU_P265G_-c-Myc in the presence of hrpG* (Fig. 2A). We have previously observed that constitutive expression of the T3S genes promotes *in planta* symptom formation (Büttner *et al*., 2004; 2007; Lorenz and Büttner, 2009). The partial complementation of the *hrcU* mutant phenotype by HrcU_P265A_-c-Myc is in agreement with the finding that this HrcU mutant derivative is partially cleaved (see Fig. 1C).

### HrcU cleavage is required for T3S of late substrates

Next, we analysed T3S in strains 85-10hrpG* (85*) and 85*Δ*hrcU* carrying HrcU-c-Myc or derivatives mutated in the NPTH motif. For this, bacteria were incubated in secretion medium and total-cell extracts and culture supernatants were analysed by immunoblotting. The translocon protein HrpF and the effector protein AvrBs3 (ectopically expressed from construct pDSF300) were detected in the culture supernatants of strains 85* and 85*Δ*hrcU* carrying HrcU-c-Myc or the mutant derivatives HrcU_P265A_-c-Myc, HrcU_T266A_-c-Myc and HrcU_H267A_-c-Myc respectively. However, the secretion efficiency in the presence of HrcU_P265A_-c-Myc and HrcU_H267A_-c-Myc was reduced when compared with HrcU_T266A_-c-Myc (Fig. 2B). This is in agreement with the finding that HrcU_P265A_-c-Myc and HrcU_H267A_-c-Myc were less efficiently cleaved than HrcU_T266A_-c-Myc (see above). No secretion of HrpF and AvrBs3 was observed for strains 85*Δ*hrcU* and 85*Δ*hrcU* carrying HrcU_N264A_-c-Myc (Fig. 2B).

We also analysed secretion of the early substrate HrpB2. When compared with strain 85*, increased amounts of HrpB2 were present in the culture supernatant of strain 85*Δ*hrcU* carrying HrcU-c-Myc, suggesting that ectopic expression of *hrcU-c-myc* positively affects HrpB2 secretion (Fig. 2B). Notably, HrpB2 was also present in the culture supernatant of strain 85*Δ*hrcU* carrying HrcU_N264A_-c-Myc, HrcU_P265A_-c-Myc, HrcU_T266A_-c-Myc and HrcU_H267A_-c-Myc respectively (Fig. 2B). This finding was unexpected and suggests that HrpB2 secretion can occur in the absence of efficient HrcU cleavage. Interestingly, however, HrpB2 was not detected in the culture supernatant of strain 85*Δ*hrcU* containing HrcU_P265G_-c-Myc (Fig. 2C). As we observed a similar finding for the translocon protein HrpF, we assume that the P265G exchange abolishes secretion of both early and late substrates (Fig. 2C).

**Fig. 3:**
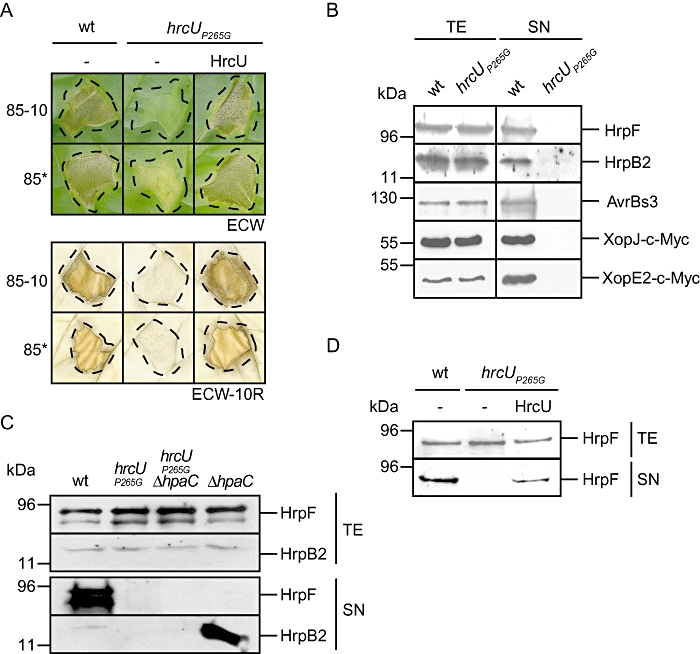
Characterization of a genomic *hrcU_P265G_* mutant. A. The P265G mutation in HrcU abolishes bacterial pathogenicity. *X. campestris* pv. *vesicatoria* strains 85-10 (wt), 85* (wt), 85-10*hrcU_P265G_* (*hrcU_P265G_*) and 85*hrcU_P265G_ (*hrcU_P265G_*) carrying the empty vector (−) or HrcU-c-Myc (HrcU) as indicated were inoculated into leaves of susceptible ECW and resistant ECW-10R pepper plants. Disease symptoms were photographed 7 dpi. For the better visualization of the HR, leaves were bleached in ethanol 2 dpi. Dashed lines indicate the infiltrated areas. B. T3S of early and late substrates is abolished in the presence of *hrcU_P265G_*. *X. campestris* pv. *vesicatoria* strains 85* (wt) and 85*hrcU_P265G_ (*hrcU_P265G_*) were incubated in secretion medium and total-cell extracts (TE) and culture supernatants (SN) were analysed by immunoblotting using antibodies specific for HrpF, HrpB2, AvrBs3 and the c-Myc epitope respectively. AvrBs3, XopJ-c-Myc and XopE2-c-Myc were encoded by corresponding expression constructs. C. HrpB2 oversecretion in the *hpaC* deletion mutant is suppressed by the genomic *hrcU_P265G_* mutation. *X. campestris* pv. *vesicatoria* strains 85* (wt), 85*hrcU_P265G_ (P265G), 85*hrcU_P265G_Δ*hpaC* (*hrcU_P265G_*Δ*hpaC*) and 85*Δ*hpaC* (Δ*hpaC*) were incubated in secretion medium. TE and SN were analysed by immunoblotting using HrpF- and HrpB2-specific antibodies. D. HrpF secretion by strain 85*hrcU_P265G_ is restored upon ectopic expression of *hrcU-c-myc*. *X. campestris* pv. *vesicatoria* strains 85* (wt) and 85*hrcU_P265G_ (*hrcU_P265G_*) carrying the empty vector (−) or encoding HrcU-c-Myc (HrcU) as indicated were incubated in secretion medium. TE and SN were analysed by immunoblotting using a HrpF-specific antibody.

To confirm these results we introduced the *hrcU_P265G_* mutation into the genome of *X. campestris* pv. *vesicatoria* strains 85-10 and 85* respectively. The resulting mutant strains 85-10*hrcU_P265G_* and 85*hrcU_P265G_ did not elicit visible disease symptoms and the HR when inoculated into leaves of susceptible and resistant pepper plants respectively (Fig. 3A). Furthermore, T3S of the translocon protein HrpF, the effector proteins AvrBs3, XopJ-c-Myc and XopE2-c-Myc (ectopically expressed from corresponding expression constructs) and HrpB2 was abolished in strain 85*hrcU_P265G_, which supports the finding that the P265G mutation in HrcU leads to a loss of protein function (Fig. 3B). Loss of efficient HrpB2 secretion was also observed in strain 85*hrcU_P265G_Δ*hpaC*, suggesting that HrpB2 oversecretion in the *hpaC* deletion mutant is suppressed in the presence of HrcU_P265G_ (Fig. 3C). The *hrcU_P265G_* mutant phenotype was restored with respect to virulence and T3S (shown for HrpF secretion) upon ectopic expression of *hrcU-c-myc* (Fig. 3A and D).

### The C-terminal domain of HrcU is essential for pathogenicity and functions *in trans*

**Fig. 4:**
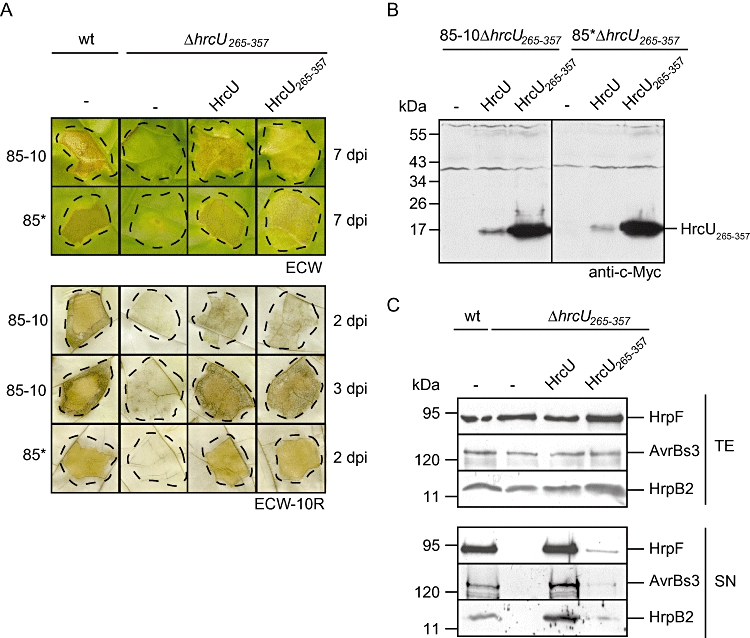
The C-terminal domain of HrcU is essential for pathogenicity. A. The Δ*hrcU_265–357_* mutant phenotype can be complemented *in trans*. *X. campestris* pv. *vesicatoria* strains 85-10 (wt), 85-10Δ*hrcU_265–357_* (Δ*hrcU_265–357_*), 85* (wt) and 85*Δ*hrcU_265–357_* (Δ*hrcU_265–357_*) carrying the empty vector (−) or expression constructs encoding HrcU-c-Myc (HrcU) and HrcU_265–357_-c-Myc (HrcU_265–357_), respectively, as indicated were inoculated into leaves of susceptible ECW and resistant ECW-10R pepper plants. Disease symptoms were photographed 7 dpi. For the better visualization of the HR, leaves were bleached in ethanol 2 or 3 dpi as indicated. Dashed lines mark the infiltrated areas. B. Protein studies with HrcU-c-Myc and HrcU_265–357_-c-Myc. *X. campestris* pv. *vesicatoria* strains 85-10Δ*hrcU_265–357_* and 85*Δ*hrcU_265–357_* carrying the empty vector (−) or expression constructs encoding HrcU-c-Myc (HrcU) and HrcU_265–357_-c-Myc (HrcU_265–357_), respectively, as indicated were grown in minimal medium A. Equal amounts of total-cell extracts were analysed by immunoblotting, using a c-Myc epitope-specific antibody. The full-length HrcU-c-Myc protein is not detectable. The dominant signal corresponds to HrcU_265–357_-c-Myc; additional signals result from unspecific binding of the antibody. C. T3S in the *hrcU_265–357_* deletion mutant. *X. campestris* pv. *vesicatoria* strains 85* (wt) and 85*Δ*hrcU_265–357_* (Δ*hrcU_265–357_*) carrying the empty vector (−) or expression constructs encoding HrcU-c-Myc (HrcU) and HrcU_265–357_-c-Myc (HrcU_265–357_), respectively, as indicated were incubated in secretion medium. Total-cell extracts (TE) and culture supernatants (SN) were analysed by immunoblotting, using antibodies specific for the translocon protein HrpF, the effector protein AvrBs3 (ectopically expressed from construct pDSF300) and HrpB2.

In addition to the NPTH motif, we studied the contribution of the C-terminal domain of HrcU (HrcU_C_, amino acids 265–357, which correspond to the predicted C-terminal HrcU cleavage product) to bacterial pathogenicity and T3S. For this, we deleted codons 265–357 of the chromosomal *hrcU* gene in *X. campestris* pv. *vesicatoria* strain 85-10. The resulting deletion mutant strain 85-10Δ*hrcU_265–357_* did not elicit disease symptoms and the HR in susceptible and resistant pepper plants, respectively, suggesting that HrcU_C_ is essential for pathogenicity (Fig. 4A). The mutant phenotype was complemented by HrcU-c-Myc whereas a partial complementation was observed when we provided a c-Myc epitope-tagged derivative of HrcU_C_*in trans* (HrcU_265–357_-c-Myc; Fig. 4A). However, HrcU_265–357_-c-Myc complemented the Δ*hrcU_265–357_* mutant phenotype in the presence of hrpG* (Fig. 4A). Immunoblot analyses of total-cell extracts from *X. campestris* pv. *vesicatoria* confirmed that HrcU-c-Myc and HrcU_265–357_-c-Myc were synthesized (Fig. 4B). As described above, we did not detect the full-length HrcU-c-Myc protein in cell extracts of *X. campestris* pv. *vesicatoria*. Furthermore, the amounts of HrcU_265–357_-c-Myc were increased when compared with the amounts of the cleavage product of HrcU-c-Myc and presumably do not reflect native protein levels (Fig. 4B). The analysis of additional expression constructs encoding HrcU_265–357_-c-Myc under control of an alternative promoter (e.g. the native *hrcU* promoter) should clarify whether the expression level of *hrcU_265–357_-c-myc* influences its ability to complement the Δ*hrcU_265–357_* mutant phenotype.

To investigate the contribution of HrcU_C_ to T3S, strains 85* and 85*Δ*hrcU_265–357_* were incubated in secretion medium and total-cell extracts and culture supernatants were analysed by immunoblotting. The translocon protein HrpF, the effector protein AvrBs3 (ectopically expressed from construct pDSF300) and the pilus assembly protein HrpB2 were detected in the culture supernatant of strain 85* but not of strain 85*Δ*hrcU_265–357_* (Fig. 4C). Wild-type levels of secretion were restored by HrcU-c-Myc, whereas HrcU_265–357_-c-Myc only partially complemented the secretion deficiency (Fig. 4C). However, as HrcU_265–357_-c-Myc restored the *in planta* phenotype of strain 85*Δ*hrcU_265–357_*, reduced levels of T3S in strain 85*Δ*hrcU_265–357_* were presumably sufficient for plant infection phenotypes (see Fig. 4A). We conclude from these findings that HrcU_C_ is crucial for T3S and pathogenicity and functions *in trans*.

### The NPTH motif of HrcU is required for the interaction with the T3S4 protein HpaC

**Fig. 5:**
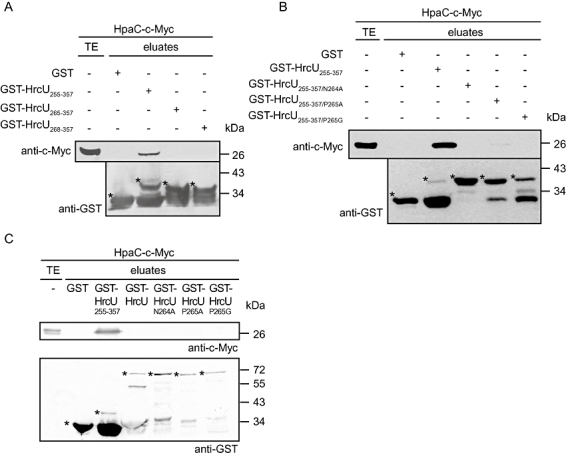
The NPTH motif of HrcU is required for the interaction with the T3S4 protein HpaC. A. Amino acids 265–357 of HrcU are not sufficient for the interaction with HpaC. GST, GST–HrcU_255–357_, GST–HrcU_265–357_ and GST–HrcU_268–357_ were immobilized on glutathione sepharose and incubated with an *E. coli* lysate containing HpaC-c-Myc. The total-cell extract (TE) and eluted proteins (eluates) were analysed by immunoblotting using c-Myc epitope- and GST-specific antibodies respectively. GST and GST fusion proteins are marked by asterisks; lower bands correspond to degradation products. B. Mutations within the NPTH motif abolish the interaction between HrcU_C_ and HpaC. GST, GST–HrcU_255–357_, GST–HrcU_255–357/N264A_, GST–HrcU_255–357/P265A_ and GST–HrcU_255–357/P265G_ were immobilized on glutathione sepharose and incubated with an *E. coli* lysate containing HpaC-c-Myc. TE and eluates were analysed as described in (A). GST and GST fusion proteins are marked by asterisks; lower bands correspond to degradation products. N264A, P265A and P265G mutations led to significantly reduced cleavage of GST–HrcU_255–357_ and thus to enhanced amounts of the full-length fusion proteins. C. HpaC-c-Myc does not bind to the full-length HrcU protein carrying mutations within the NPTH motif. GST, GST–HrcU_255–357_, GST–HrcU_N264A_, GST–HrcU_P265A_ and GST–HrcU_P265G_ were immobilized on glutathione sepharose and incubated with an *E. coli* lysate containing HpaC-c-Myc. TE and eluates were analysed as described in (A). GST and GST fusion proteins are marked by asterisks; lower bands correspond to degradation products.

We have previously shown that the T3S4 protein HpaC interacts with a GST–HrcU_255–357_ fusion protein (Lorenz *et al*., 2008b). To investigate whether the interaction depends on the NPTH motif (amino acids 264–268) of HrcU, we generated additional expression constructs encoding GST–HrcU_265–357_, which lacks the conserved asparagine residue, and GST–HrcU_268–357_, which is deprived of the complete NPTH motif. For protein–protein interaction studies, GST, GST–HrcU_255–357_, GST–HrcU_265–357_ and GST–HrcU_268–357_ were synthesized in *E. coli*, immobilized on glutathione sepharose and incubated with an *E. coli* lysate containing HpaC-c-Myc. Eluted proteins were analysed by immunoblotting using a c-Myc epitope-specific antibody. Figure 5A shows that HpaC-c-Myc was detected in the eluate of GST–HrcU_255–357_ as expected but not of GST, GST–HrcU_265–357_ and GST–HrcU_268–357_. We also performed interaction studies with GST–HrcU_255–357_ derivatives carrying single amino acid substitutions of the conserved asparagine and proline residues (N264A, P265A and P265G) of the NPTH motif. When GST–HrcU_255–357/N264A_, GST–HrcU_255–357/P265A_ and GST–HrcU_255–357/P265G_ were immobilized on glutathione sepharose and incubated with HpaC-c-Myc, HpaC-c-Myc was not detected in the eluates, suggesting that mutations of N264 and P265 abolish the efficient binding of HpaC to HrcU_C_ (Fig. 5B).

We also analysed the influence of N264A, P265A and P265G mutations on the HrcU_C_–HpaC interaction in the context of the full-length HrcU protein. As described above, HpaC does not interact with the full-length HrcU protein, yet, it could not be excluded that the N264A, P265A and P265G mutations in HrcU lead to an alteration of the protein conformation that is permissive for binding of HpaC. However, when GST–HrcU_255–357_, GST–HrcU, GST–HrcU_N264A_, GST–HrcU_P265A_ and GST–HrcU_P265G_ were immobilized on glutathione sepharose and incubated with HpaC-c-Myc, we detected HpaC-c-Myc in the eluate of GST–HrcU_255–357_ as expected but not of GST–HrcU and mutant derivatives thereof (Fig. 5C). We have previously reported that GST–HrcU can be stably synthesized in *E. coli* and that sufficient amounts of the protein are present in the soluble fraction (Lorenz *et al*., 2008b). We observed similar findings for GST–HrcU_255–357_ and GST–HrcU derivatives carrying point mutations in the NPTH motif (Fig. S1; data not shown).

### The NPTH motif contributes to the interaction between HrcU_C_ and HrpB2

**Fig. 6:**
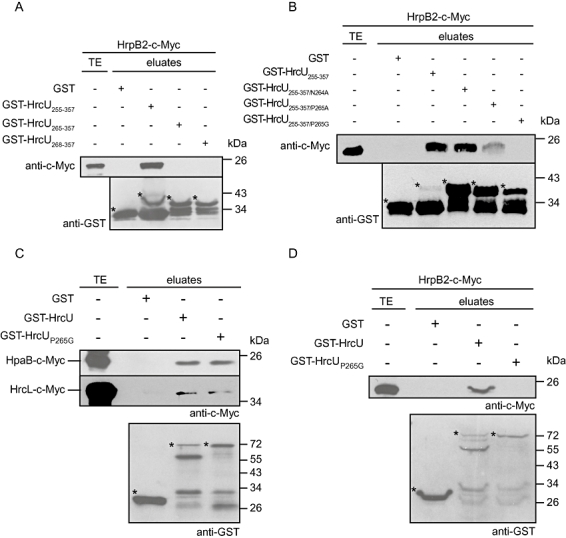
The NPTH motif of HrcU contributes to the interaction between HrcU_C_ and HrpB2. A. Amino acids 265–357 of HrcU are not sufficient for the interaction with HrpB2. GST, GST–HrcU_255–357_, GST–HrcU_265–357_ and GST–HrcU_268–357_ were immobilized on glutathione sepharose and incubated with an *E. coli* lysate containing HrpB2-c-Myc. The total-cell extract (TE) and eluted proteins (eluates) were analysed by immunoblotting using c-Myc epitope- and GST-specific antibodies respectively. GST and GST fusion proteins are marked by asterisks; lower bands correspond to degradation products. B. The P265G exchange abolishes the interaction between HrcU_C_ and HrpB2. GST, GST–HrcU_255–357_, GST–HrcU_255–357/N264A_, GST–HrcU_255–357/P265A_ and GST–HrcU_255–357/P265G_ were immobilized on glutathione sepharose and incubated with an *E. coli* lysate containing HrpB2-c-Myc. TE and eluates were analysed as described in (A). GST and GST fusion proteins are marked by asterisks; lower bands correspond to degradation products. N264A, P265A and P265G mutations led to significantly reduced cleavage of GST–HrcU_255–357_ and thus to enhanced amounts of the full-length fusion proteins. C. The P265G exchange in HrcU does not affect binding of both HpaB and HrcL to HrcU. GST, GST–HrcU and GST–HrcU_P265G_ were immobilized on glutathione sepharose and incubated with *E. coli* lysates containing HpaB-c-Myc and HrcL-c-Myc respectively. TE and eluates were analysed as described in (A). GST and GST fusion proteins are marked by asterisks; lower bands correspond to degradation products. One representative blot probed with the GST-specific antibody is shown. D. GST–HrcU_P265G_ does not interact with HrpB2. GST, GST–HrcU and GST–HrcU_P265G_ were immobilized on glutathione sepharose and incubated with an *E. coli* lysate containing HrpB2-c-Myc. TE and eluates were analysed as described in (A). GST and GST fusion proteins are marked by asterisks; lower bands correspond to degradation products.

In addition to HpaC, GST–HrcU_255–357_ also interacts with a C-terminally c-Myc epitope-tagged derivative of the early T3S substrate HrpB2 (Lorenz *et al*., 2008b). To investigate whether the NPTH motif of HrcU contributes to the interaction between HrcU_C_ and HrpB2, we performed additional pull-down assays with GST or GST–HrcU derivatives as described above. When GST–HrcU fusion proteins were immobilized on glutathione sepharose and incubated with HrpB2-c-Myc, we detected HrpB2-c-Myc in the eluate of GST–HrcU_255–357_ as expected but not in the eluates of GST–HrcU_265–357_ and GST–HrcU_268–357_ (Fig. 6A; Lorenz *et al*., 2008b). The presence of N264A and P265A point mutations in GST–HrcU_255–357_, respectively, did not significantly affect the binding of HrpB2-c-Myc (Fig. 6B). In contrast, HrpB2-c-Myc was not detected in the eluate of GST–HrcU_255–357/P265G_ indicating that the P265G exchange abolishes the stable interaction between GST–HrcU_255–357_ and HrpB2 (Fig. 6B).

To investigate whether the P265G mutation also prevents binding of additional interaction partners of HrcU, we performed interaction studies with GST–HrcU_P265G_ and C-terminally c-Myc epitope-tagged derivatives of the general T3S chaperone HpaB and the predicted regulator of the ATPase, HrcL. HpaB and HrcL were previously shown to interact with GST–HrcU but not with GST–HrcU_255–357_ (Lorenz and Büttner, 2009). HpaB-c-Myc and HrcL-c-Myc were detected in the eluates of GST–HrcU and GST–HrcU_P265G_ but not of GST alone, suggesting that the P265G exchange in HrcU did not affect the interaction with both HpaB and HrcL (Fig. 6C). As an additional control, we incubated immobilized GST–HrcU_P265G_ with HrpB2-c-Myc. Figure 6D shows that HrpB2-c-Myc was not detectable in the eluate of GST–HrcU_P265G_, which confirms the above finding that the P265G mutation abolishes the interaction between HrcU_C_ and HrpB2. In this context it is of interest to note that HrcU_P265G_ did not promote secretion of HrpB2. It is therefore conceivable that the interaction between HrcU_C_ and HrpB2 is required for efficient HrpB2 secretion (see above, Fig. 2).

### A point mutation (Y318D) in HrcU_C_ suppresses the *hpaC* mutant phenotype

**Fig. 7:**
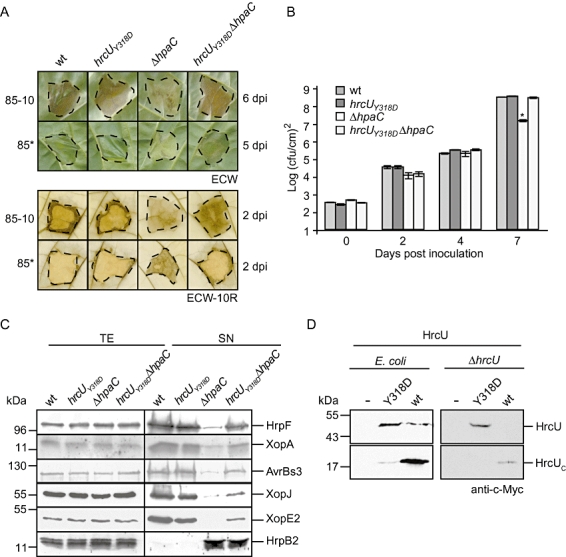
The Y318D mutation in HrcU suppresses the *hpaC* mutant phenotype and affects HrcU cleavage. A. Infection studies with *hrcU* wild-type and *hrcU_Y318D_* mutant strains. *X. campestris* pv. *vesicatoria* strains 85-10 (wt), 85* (wt), 85-10*hrcU_Y318D_* (*hrcU_Y318D_*), 85*hrcU_Y318D_ (*hrcU_Y318D_*), 85-10Δ*hpaC* (Δ*hpaC*), 85*Δ*hpaC* (Δ*hpaC*), 85-10*hrcU_Y318D_*Δ*hpaC* (*hrcU_Y318D_*Δ*hpaC*) and 85*hrcU_Y318D_Δ*hpaC* (*hrcU_Y318D_*Δ*hpaC*) were inoculated into leaves of susceptible ECW and resistant ECW-10R pepper plants. Disease symptoms were photographed 5 and 6 dpi as indicated. For the better visualization of the HR, leaves were bleached in ethanol 2 dpi. Dashed lines mark the infiltrated areas. B. *In planta* growth of *hrcU_Y318D_* mutants. *X. campestris* pv. *vesicatoria* strains 85-10 (wt), 85-10*hrcU_Y318D_* (*hrcU_Y318D_*), 85-10Δ*hpaC* (Δ*hpaC*) and 85-10*hrcU_Y318D_*Δ*hpaC* (*hrcU_Y318D_*Δ*hpaC*) were inoculated into leaves of susceptible ECW pepper plants and bacterial growth was analysed over a period of 8 days. Values are the mean of three samples from three plants. Error bars represent standard deviations. The asterisk indicates a significant difference to the wild-type strain with *P* < 0.005 based on the results of an unpaired Student's *t-*test. C. T3S assays with *hrcU_Y318D_* mutants. Strains 85* (wt), 85*hrcU_Y318D_ (*hrcU_Y318D_*), 85*Δ*hpaC* (Δ*hpaC*) and 85*hrcU_Y318D_Δ*hpaC* (*hrcU_Y318D_*Δ*hpaC*) were incubated in secretion medium. Total-cell extracts (TE) and culture supernatants (SN) were analysed by immunoblotting using antibodies specific for the putative translocon proteins HrpF and XopA, the effector protein AvrBs3, the pilus assembly protein HrpB2 and the c-Myc epitope. AvrBs3, XopJ-c-Myc and XopE2-c-Myc were encoded by corresponding expression constructs. D. The Y318D mutation in HrcU affects proteolytic cleavage. Equal amounts of total-cell extracts from *X. campestris* pv. *vesicatoria* strain 85*Δ*hrcU* (Δ*hrcU*) and *E. coli* carrying the empty vector (−) or encoding HrcU-c-Myc (wt) and HrcU_Y318D_-c-Myc (Y318D), respectively, as indicated were analysed by immunoblotting using a c-Myc epitope-specific antibody.

It was previously reported that the phenotype of T3S4 mutants from animal pathogenic bacteria can be suppressed upon introduction of point mutations into the C-terminal domain of FlhB/YscU family members (Kutsukake *et al*., 1994; Williams *et al*., 1996; Edqvist *et al*., 2003; Wood *et al*., 2008; Zarivach *et al*., 2008). To test this for *X. campestris* pv. *vesicatoria*, we introduced a point mutation (Y318D) into the chromosomal *hrcU* genes of strains 85-10 and 85-10Δ*hpaC*, respectively, which led to an exchange of the tyrosine residue at amino acid position 318 of HrcU by aspartic acid. Equivalent mutations in the C-terminal domains of YscU (YscU_Y317D_) and FlhB (FlhB_Y323D_) were shown to suppress the phenotypes of mutants deleted in the T3S4 genes *yscP* and *fliK* respectively (Kutsukake *et al*., 1994; Minamino and MacNab, 2000a; Edqvist *et al*., 2003; Wood *et al*., 2008). When *X. campestris* pv. *vesicatoria hrcU* wild-type and *hrcU_Y318D_* mutant strains were inoculated into leaves of susceptible ECW and resistant ECW-10R pepper plants, respectively, strain 85-10*hrcU_Y318D_* induced disease symptoms and the HR similarly to the wild type whereas strain 85-10Δ*hpaC* led to significantly reduced symptoms as expected (Fig. 7A; Büttner *et al*., 2006). The double mutant 85-10*hrcU_Y318D_*Δ*hpaC* induced wild-type disease symptoms, suggesting that HrcU_Y318D_ suppresses the *hpaC* mutant phenotype in susceptible plants (Fig. 7A). Furthermore, HrcU_Y318D_ partially restored the HR induction by strain 85-10*hrcU_Y318D_*Δ*hpaC* in resistant ECW-10R plants. However, a wild-type HR was observed for the hrpG* derivative 85*hrcU_Y318D_Δ*hpaC* (Fig. 7A).

We also analysed *in planta* bacterial growth of strains 85-10, 85-10Δ*hpaC*, 85-10*hrcU_Y318D_* and 85-10*hrcU_Y318D_*Δ*hpaC* in susceptible ECW pepper plants. As described earlier, bacterial counts of strain 85-10Δ*hpaC* were significantly reduced 8 days post inoculation (dpi) when compared with the wild-type strain, suggesting that HpaC contributes to bacterial multiplication at later stages of the infection (Fig. 7B; Büttner *et al*., 2006). Strain 85-10*hrcU_Y318D_*Δ*hpaC* grew similarly to strain 85-10, which is in agreement with the observation that HrcU_Y318D_ suppresses the *hpaC* mutant phenotype with respect to disease symptoms (Fig. 7B).

### HrcU_Y318D_ restores secretion of translocon and effector proteins but does not affect HrpB2 oversecretion in the *hpaC* deletion mutant

In addition to infection experiments, we performed T3S assays with strains 85*, 85*hrcU_Y318D_, 85*Δ*hpaC* and 85*hrcU_Y318D_Δ*hpaC*. Figure 7C shows that comparable amounts of the putative translocon proteins HrpF and XopA and the effector proteins AvrBs3, XopJ-c-Myc and XopE2-c-Myc (encoded by corresponding expression constructs) were secreted by strains 85* and 85*hrcU_Y318D_, respectively, whereas secretion of these proteins by strain 85*Δ*hpaC* was severely reduced as expected (Büttner *et al*., 2006). Efficient secretion was restored in strain 85*hrcU_Y318D_Δ*hpaC*, suggesting that HrcU_Y318D_ activates secretion of late T3S substrates including translocon and effector proteins in the absence of HpaC (Fig. 7C).

In addition to translocon and effector proteins, we analysed secretion of HrpB2, which is secreted in small amounts (at the detection limit of the HrpB2-specific antibody) by the wild-type strain and oversecreted by the *hpaC* deletion mutant (Fig. 7C; Rossier *et al*., 2000; Lorenz *et al*., 2008b). Interestingly, oversecretion of HrpB2 was also observed for strain 85*hrcU_Y318D_Δ*hpaC*. Thus, HrcU_Y318D_ suppresses the *hpaC* mutant phenotype with respect to disease symptoms and T3S of late substrates but does not affect secretion of HrpB2 (Fig. 7C). This finding was unexpected and implies that secretion of early (HrpB2) and late (translocon and effector proteins) T3S substrates in *X. campestris* pv. *vesicatoria* is controlled by different mechanisms that can be uncoupled.

We also investigated whether the Y318D mutation affects HrcU cleavage. For this, we generated an expression construct encoding HrcU_Y318D_-c-Myc and analysed the protein in *X. campestris* pv. *vesicatoria* strain 85*Δ*hrcU* by immunoblotting. We detected the full-length HrcU_Y318D_-c-Myc protein and the C-terminal cleavage product; however, the amounts of the cleavage product were significantly reduced when compared with the wild-type HrcU-c-Myc (Fig. 7D). A similar difference in proteolytic cleavage was observed in *E. coli* (Fig. 7D). As both c-Myc epitope-tagged HrcU derivatives were only synthesized at low levels in *E. coli*, we did not detect full-length HrcU-c-Myc and the C-terminal cleavage product of HrcU_Y318D_-c-Myc (Fig. 7D). Taken together, we conclude from these findings that the Y318D exchange in HrcU prevents efficient HrcU cleavage but activates secretion of late substrates in the absence of HpaC.

### The Y318D mutation affects binding of both HrpB2 and HpaC to HrcU_C_

**Fig. 8:**
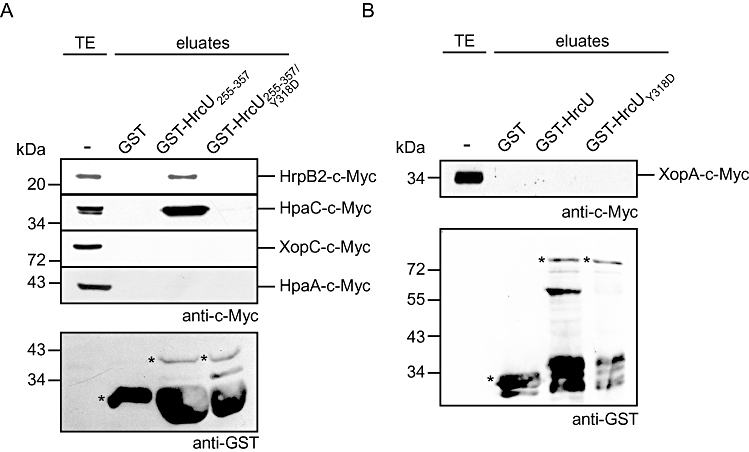
The Y318D mutation abolishes the interaction between the C-terminal region of HrcU and both HrpB2 and HpaC. A. GST–HrcU_255–357/Y318D_ does not interact with HrpB2, HpaC and T3S substrates. GST, GST–HrcU_255–357_ and GST–HrcU_255–357/Y318D_ were immobilized on glutathione sepharose and incubated with *E. coli* lysates containing HrpB2-c-Myc, HpaC-c-Myc, XopC-c-Myc and HpaA-c-Myc respectively. Total-cell extracts (TE) and eluted proteins (eluates) were analysed by immunoblotting, using c-Myc- and GST-specific antibodies. Asterisks mark GST and GST fusion proteins; lower bands correspond to degradation products. One representative blot probed with the GST-specific antibody is shown. B. HrcU_Y318D_ does not interact with the putative translocon protein XopA. GST, GST–HrcU and GST–HrcU_Y318D_ were immobilized on glutathione sepharose and incubated with XopA-c-Myc. TE and eluates were analysed as described in (A). Asterisks mark GST and GST fusion proteins; lower bands correspond to degradation products.

As HrcU_Y318D_ presumably mimics a protein conformation that is permissive for the secretion of late substrates, we investigated a possible influence of the Y318D mutation on the interaction of HrcU_C_ with HrpB2 and HpaC. For this, GST, GST–HrcU_255–357_ and GST–HrcU_255–357/Y318D_ were immobilized on glutathione sepharose and incubated with HrpB2-c-Myc and HpaC-c-Myc respectively. Figure 8A shows that HrpB2-c-Myc and HpaC-c-Myc co-eluted with GST–HrcU_255–357_ as expected but were not detectable in the eluate of GST–HrcU_255–357/Y318D_, suggesting that the Y318D mutation prevents the stable binding of both HrpB2 and HpaC to HrcU_C_. Given the finding that HrpB2 is oversecreted by strain 85*hrcU_Y318D_Δ*hpaC*, it is conceivable that the interaction of HrcU_C_ and HrpB2 is not required for efficient HrpB2 secretion after the substrate specificity switch.

To date, HrpB2 is the only known T3S substrate that was shown to interact with HrcU_C_ (Lorenz *et al*., 2008b). To investigate whether a potential binding of T3S substrates to HrcU is restricted to a certain protein conformation that is mimicked in the presence of HrcU_Y318D_, we immobilized GST–HrcU_255–357_ and GST–HrcU_255–357/Y318D_ on glutathione sepharose and incubated both proteins with C-terminally c-Myc epitope-tagged derivatives of the effector proteins HpaA and XopC respectively. Immunoblot analyses revealed that HpaA-c-Myc and XopC-c-Myc were not detectable in the eluates, suggesting that they did not stably interact with HrcU and HrcU_Y318D_ (Fig. 8A). Similarly, we did not detect a c-Myc epitope-tagged derivative of the putative translocon protein XopA in the eluates of GST–HrcU and GST–HrcU_Y318D_ (Fig. 8B).

## Discussion

In this study, we describe novel mechanisms underlying the orchestration of T3S substrate specificity switching in a plant pathogenic bacterium. We investigated the role of the inner membrane protein HrcU and the T3S4 protein HpaC from *X. campestris* pv. *vesicatoria* during T3S and provide experimental evidence that HpaC binds to the conserved NPTH motif of HrcU, which is the predicted cleavage site. The analysis of HrcU mutant derivatives carrying single amino acid exchanges within the NPTH motif revealed that mutations of T266 and H267, respectively, only slightly affect HrcU cleavage whereas the N264A exchange abolishes detectable cleavage. For FlhB/YscU family members from animal pathogenic bacteria it was previously reported that cleavage is an autocatalytic process that involves cyclization of the conserved asparagine residue of the NPTH motif (Ferris *et al*., 2005; Deane *et al*., 2008; Zarivach *et al*., 2008; Lountos *et al*., 2009; Wiesand *et al*., 2009). In agreement with this model, mutation of the asparagine residue of the NPTH motif prevents cleavage not only of HrcU from *X. campestris* pv. *vesicatoria* but also of the homologous YscU, EscU and FlhB proteins from animal pathogenic bacteria (Lavander *et al*., 2002; Fraser *et al*., 2003; Sorg *et al*., 2007; Riordan and Schneewind, 2008; Zarivach *et al*., 2008; Björnfot *et al*., 2009; Smith *et al*., 2009; Wiesand *et al*., 2009). Exchange of the conserved proline residue P265 of HrcU by alanine led to a significant reduction of HrcU cleavage whereas the P265G mutation resulted in a complete loss of detectable cleavage (Fig. 1). A similar difference in cleavage was described for P264A and P264G mutant derivatives of the HrcU homologue YscU from *Yersinia* (Wiesand *et al*., 2009). Because autocatalytic cleavage of YscU depends on the positioning of the carbonyl group of the asparagine residue at position 263, the efficiency of the cleavage is presumably influenced by the amino acid residue at position 264 (Wiesand *et al*., 2009). Given the finding that YscU homologues share significant structural similarities (Deane *et al*., 2008; Zarivach *et al*., 2008; Lountos *et al*., 2009; Wiesand *et al*., 2009), a similar scenario might explain the difference in proteolytic cleavage of HrcU_P265A_ and HrcU_P265G_.

Complementation studies with HrcU point mutants from *X. campestris* pv. *vesicatoria* revealed that loss of detectable HrcU cleavage correlates with a loss of bacterial pathogenicity (Fig. 2), which was confirmed for HrcU_P265G_ by the analysis of a genomic *hrcU_P265G_* mutation (Fig. 3). This is an important experimental control because it was previously observed that the effects of point mutations in YscU from *Yersinia* might vary depending on whether YscU derivatives are provided *in cis* or *in trans* (Sorg *et al*., 2007; Björnfot *et al*., 2009). Taken together, we conclude from the analysis of HrcU point mutant derivatives that cleavage of HrcU is essential for the interaction of the bacteria with the plant. Notably, however, we also observed that HrcU_C_ can function *in trans*, suggesting that it is not the cleavage event *per se* but rather the result of the cleavage which is required for pathogenicity (Fig. 4). A similar finding was previously reported for a HrcU homologue from *Helicobacter pylori* (Wand *et al*., 2006).

The N264A and P265A mutations in HrcU did not only lead to a loss of pathogenicity but also to a reduction in T3S of translocon and effector proteins. This is in contrast to the finding that the equivalent N263A exchange in the HrcU homologue YscU from *Yersinia* spp. abolished secretion of translocon but not of effector proteins (Sorg *et al*., 2007). It was therefore proposed that YscU cleavage is required to switch the T3S substrate specificity to translocon but not to effector proteins. The mechanisms underlying control of T3S substrate specificity switching might therefore vary in *Yersinia* spp. and *X. campestris* pv. *vesicatoria*.

**Fig. 9:**
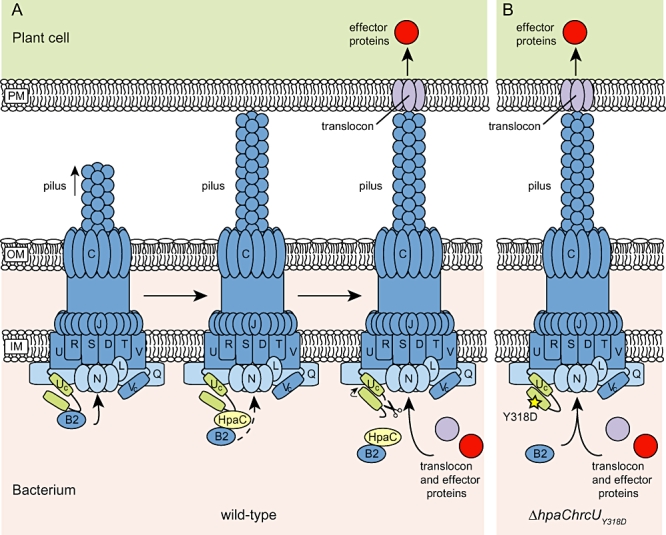
Model of the molecular mechanisms underlying the HpaC-HrcU_C_-mediated substrate specificity switch in *X. campestris* pv. *vesicatoria*. A. HpaC controls secretion of early and late T3S substrates. The T3S system of *X. campestris* pv. *vesicatoria* consists of approximately 20 components, eleven of which (abbreviated with single letters) are designated Hrc (Hrp conserved) and presumably constitute the core components of the membrane-spanning secretion apparatus. Cytoplasmic components of the T3S apparatus are shown in light blue, the C-terminal domain of HrcU in green. During the initial step of T3S the early T3S substrate HrpB2 (abbreviated B2), which is required for pilus assembly, interacts with the C-terminal cytoplasmic domain of HrcU and is secreted. The efficient secretion of HrpB2 is inhibited upon binding of the T3S4 protein HpaC to HrpB2 and/or to HrcU_C_. The cleavage of HrcU at the conserved NPTH motif and a conformational change in HrcU_C_ lead to the release of HrcU_C_-bound HpaC and HrpB2 and activate the secretion of late substrates including translocon and effector proteins. Dashed lines refer to reduced secretion of HrpB2, the arrow next to HrcU_C_ to the predicted conformational change. B. The Y318D mutation in HrcU_C_ activates secretion of late substrates in the absence of HpaC. The Y318D mutation presumably leads to a conformational change in HrcU_C_, which allows the efficient secretion of late substrates but leads to reduced cleavage of HrcU and also abolishes the interaction between HrcU_C_ and HrpB2. Secretion of HrpB2 is not affected by the Y318D exchange in HrcU.IM, inner membrane; OM, outer membrane; PM, plasma membrane of the host cell.

In contrast to translocon and effector proteins, HrpB2 was efficiently secreted by HrcU cleavage mutants carrying alanine substitutions within the NPTH motif (Fig. 2). For yet unknown reasons, ectopic expression of *hrcU* under control of the *lac* promoter in a *hrcU* deletion mutant background led to increased HrpB2 secretion that was independent of HrcU cleavage. This implies that HrpB2 secretion is controlled by the amounts of HrcU and occurs prior to HrcU cleavage, which is in agreement with the notion that HrpB2 is an early substrate of the T3S system (Fig. 9). We previously reported that HrpB2 interacts with the C-terminal domain of HrcU (Lorenz *et al*., 2008b). Here, we show that HrpB2 does not stably interact with GST–HrcU deletion derivatives lacking the NPTH motif or carrying a P265G mutation (shown in the context of both GST–HrcU_255–357_ and GST–HrcU; Fig. 6). In contrast, N264A and P265A mutations in GST–HrcU_255–357_ did not significantly affect the interaction between HrcU_C_ and HrpB2. It is conceivable that binding of HrpB2 depends on a certain conformation of HrcU_C_ in or around the NPTH motif that is altered in P265G but not in N264A or P265A HrcU mutant derivatives. However, the P265G mutation presumably did not lead to a complete misfolding of HrcU because the interaction with the putative ATPase regulator HrcL and the general T3S chaperone HpaB was not affected. Notably, HrcU_P265G_ did not promote secretion of HrpB2, which is in contrast to the mutant derivatives HrcU_N264A_ and HrcU_P265A_ (Fig. 2). It is therefore possible that the interaction between HrpB2 and HrcU_C_ is required for the efficient secretion of HrpB2 during the early stage of the T3S process, i.e. prior to HrcU cleavage (Fig. 9).

The results of our protein–protein interaction studies showed that mutations in the NPTH motif of HrcU did not only affect the HrcU_C_–HrpB2 interaction but also the binding of HpaC to HrcU_C_ (Fig. 5). To our knowledge, this is the first experimental evidence that T3S4 proteins and T3S substrates compete for the same binding site in the C-terminal domains of FlhB/YscU family members. It remains to be investigated whether HpaC from *X. campestris* pv. *vesicatoria* prevents the efficient secretion of HrpB2 by blocking its access to HrcU_C_. Alternatively, given the finding that HpaC interacts with HrpB2 (Lorenz *et al*., 2008b), a direct interaction of both proteins in the bacterial cytoplasm might interfere with efficient HrpB2 secretion (Fig. 9). The latter hypothesis would explain the inability of HrcU_Y318D_ to restore wild-type levels of HrpB2 secretion in the absence of HpaC (see below; Figs 7 and 9). As the Y318D mutation in HrcU suppressed the *hpaC* mutant phenotype with respect to secretion of late substrates and disease symptom formation (Fig. 7), we conclude that the elevated levels of secreted HrpB2 in the *hrcU_Y318D_*Δ*hpaC* double mutant are not detrimental for bacterial pathogenicity.

HrpB2 shares limited sequence similarity with predicted inner rod proteins from animal pathogenic bacteria that presumably assemble at the base of the needle (Sukhan *et al*., 2003; Marlovits *et al*., 2006). It was previously reported that the inner rod protein YscI from *Yersinia* spp. is oversecreted in the absence of the T3S4 protein YscP (Wood *et al*., 2008). This is reminiscent of our finding that HrpB2 is oversecreted in the *hpaC* deletion mutant. Notably, however, wild-type secretion levels of YscI in the *yscP* mutant can be restored upon introduction of the point mutation Y317D into YscU (equivalent to mutation Y318D in HrcU from *X. campestris* pv. *vesicatoria*). As YscU_Y317D_ also restores the wild-type phenotype in the *yscP* mutant (Edqvist *et al*., 2003), the control mechanisms underlying secretion of YscI and late T3S substrates from *Yersinia* spp. are presumably linked. Notably, this is in contrast to our finding that HrcU_Y318D_ activates secretion of late substrates without affecting HrpB2 secretion. Secretion of HrpB2 and late substrates from *X. campestris* pv. *vesicatoria* is therefore presumably controlled by independent mechanisms that can be uncoupled. Given the possibility that HrpB2 secretion is directly controlled by HpaC, we will localize HpaC binding sites in HrpB2 and analyse their contribution to the control of HrpB2 secretion in future studies.

The identification of the Y318D exchange in HrcU_C_ as an extragenic suppressor mutation of the *hpaC* mutant phenotype is reminiscent of the finding that point mutations in the C-terminal domains of FlhB and YscU suppress the phenotypes of T3S4 mutants from *S. typhimurium* and *Y. pseudotuberculosis*, respectively (Williams *et al*., 1996; Edqvist *et al*., 2003; Wood *et al*., 2008), and confirms the predicted role of the C-terminal domain of HrcU during the substrate specificity switch. It is tempting to speculate that HrcU_Y318D_ mimics a protein conformation of HrcU that allows the efficient secretion of late substrates including translocon and effector proteins in the absence of the T3S4 protein HpaC. Interestingly, the Y318D mutation in HrcU did not only suppress the *hpaC* mutant phenotype but also led to a significant reduction in proteolytic cleavage of HrcU and abolished stable binding of both HpaC and HrpB2 to HrcU_C_. Comparative sequence and crystal structure analyses of HrcU homologues from animal pathogenic bacteria revealed that the tyrosine residues corresponding to Y318 of HrcU are part of a conserved LARXLY amino acid motif, which is positioned in the vicinity of the PTH loop (Deane *et al*., 2008; Zarivach *et al*., 2008). Mutations in the LARXLY motif can therefore alter the orientation of the PTH loop, which might explain the reduced proteolytic cleavage and impaired binding of HrcU_Y318D_ to HpaC and HrpB2. The finding that HrcU_Y318D_ is less efficiently cleaved but suppresses the *hpaC* mutant phenotype suggests that secretion of late substrates can occur in the absence of efficient cleavage of HrcU and thus supports the notion that the T3S substrate specificity switch rather depends on a certain protein conformation of HrcU than on the cleavage event itself. Furthermore, we conclude from our data that the T3S substrate specificity switch that is mimicked in the presence of HrcU_Y318D_ leads to the release of HrcU_C_-bound HpaC and HrpB2 (Fig. 9). As the Y318D exchange did not alter HrpB2 oversecretion in the *hpaC* deletion mutant, we assume that the interaction between HrcU_C_ and HrpB2 is dispensable for efficient HrpB2 secretion during later stages of the T3S process, i.e. after the T3S substrate specificity switch (Fig. 9). It remains to be investigated whether the switch and thus the predicted conformational change in HrcU_C_ expose additional substrate acceptor sites at the inner membrane that could promote the entry of HrpB2 into the T3S channel in the absence of the HrcU_C_–HrpB2 interaction. In future studies, we therefore aim at the identification of T3S substrate docking sites in conserved components of the T3S system that are associated with the inner bacterial membrane.

## Experimental procedures

### Bacterial strains and growth conditions

Bacterial strains and plasmids used in this study are listed in Table 1. *E. coli* cells were grown at 37°C in lysogeny broth (LB) or Super medium (Qiagen, Hilden, Germany). *X. campestris* pv. *vesicatoria* strains were cultivated at 30°C in nutrient-yeast-glycerol (NYG) medium (Daniels *et al*., 1984) or in minimal medium A (Ausubel *et al*., 1996) supplemented with sucrose (10 mM) and casamino acids (0.3%). Plasmids were introduced into *E. coli* by electroporation and into *X. campestris* pv. *vesicatoria* by conjugation, using pRK2013 as a helper plasmid in triparental matings (Figurski and Helinski, 1979). Antibiotics were added to the media at the following final concentrations: ampicillin, 100 µg ml^−1^; kanamycin, 25 µg ml^−1^; rifampicin, 100 µg ml^−1^; spectinomycin, 100 µg ml^−1^; gentamicin, 7.5 µg ml^−1^.

**Table 1:** Bacterial strains and plasmids used in this study

	Relevant characteristics	Reference or source
*X. campestris* pv. *vesicatoria*		
85-10	Pepper-race 2; wild type; Rif^r^	Canteros (1990); Kousik and Ritchie (1998)
85-10Δ*hrcU*	85-10 derivative deleted in codons 3–351 of *hrcU*	This study
85-10Δ*hrcU_265–357_*	85-10 derivative deleted in codons 265–357 of *hrcU*	This study
85-10*hrcU_Y318D_*	*hrcU_Y318D_* mutant derivative of strain 85-10	This study
85-10*hrcU_P265G_*	*hrcU_P265G_* mutant derivative of strain 85-10	This study
85-10Δ*hpaC*	*hpaC* deletion mutant of strain 85-10	Büttner *et al*. (2006)
85-10*hrcU_Y318D_*Δ*hpaC*	*hrcU_Y318D_* mutant derivative of strain 85-10Δ*hpaC*	This study
85*	85-10 derivative containing the hrpG* mutation	Wengelnik *et al*. (1999)
85*Δ*hrcU*	85* derivative deleted in codons 3–351 of *hrcU*	This study
85*Δ*hrcU_265–357_*	85* derivative deleted in codons 265–357 of *hrcU*	This study
85*hrcU_Y318D_	*hrcU_Y318D_* mutant derivative of strain 85*	This study
85*hrcU_P265G_	*hrcU_P265G_* mutant derivative of strain 85*	This study
85*Δ*hpaC*	*hpaC* deletion mutant of strain 85*	Büttner *et al*. (2006)
85*hrcU_Y318D_Δ*hpaC*	*hrcU_Y318D_* mutant derivative of strain 85*Δ*hpaC*	This study
85*hrcU_P265G_Δ*hpaC*	*hrcU_P265G_* mutant derivative of strain 85*Δ*hpaC*	This study
*E. coli*		
BL21 (DE3)	F^-^*omp*T *hsd*S_B_ (r_B_^-^ m_B_^-^) *gal dcm* (DE3)	Stratagene, Heidelberg, Germany
DH5α	F^-^*recA hsdR17(r_k_^-^,m_k_^+^) Φ80dlacZ*Δ*M15*	Bethesda Research Laboratories, Bethesda, MD
DH5αλpir	F^-^*recA hsdR17(r_k_^-^,m_k_^+^) Φ80dlacZ*Δ*M15 [λpir]*	Ménard *et al*. (1993)
Plasmids		
pBlueskript(II) KS	Phagemid, pUC derivative; Ap^r^	Stratagene
pBRM	Golden Gate-compatible derivative of pBBR1MCS-5	Szczesny *et al*. (2010)
pBRMhrcU	pBRM derivative encoding HrcU-c-Myc	This study
pBRMhrcU_Y318D_	pBRM derivative encoding HrcU_Y318D_-c-Myc	This study
pBRMhrcU_N264A_	pBRM derivative encoding HrcU_N264A_-c-Myc	This study
pBRMhrcU_P265A_	pBRM derivative encoding HrcU_P265A_-c-Myc	This study
pBRMhrcU_T266A_	pBRM derivative encoding HrcU_T266A_-c-Myc	This study
pBRMhrcU_H267A_	pBRM derivative encoding HrcU_H267A_-c-Myc	This study
pBRMhrcU_P265G_	pBRM derivative encoding HrcU_P265G_-c-Myc	This study
pBRMhrcU_265–357_	pBRM derivative encoding HrcU_265–357_-c-Myc	This study
pBRMhrcU_206–357_	pBRM derivative encoding HrcU_206–357_-c-Myc	This study
pBRMhrcU_206–357/N264A_	pBRM derivative encoding HrcU_206–357/N264A_-c-Myc	This study
pBRMhrcU_206–357/P265A_	pBRM derivative encoding HrcU_206–357/P265A_-c-Myc	This study
pBRMhrcU_206–357/T266A_	pBRM derivative encoding HrcU_206–357/T266A_-c-Myc	This study
pBRMhrcU_206–357/H267A_	pBRM derivative encoding HrcU_206–357/H267A_-c-Myc	This study
pBRMxopA	pBRM derivative encoding XopA-c-Myc	This study
pBRMxopE2	pBRM derivative encoding XopE2-c-Myc	This study
pBRMxopJ	pBRM derivative encoding XopJ-c-Myc	This study
pDSK602	Broad-host-range vector; contains triple *lacUV5* promoter; Sm^r^	Murillo *et al*. (1994)
pDSK604	Derivative of pDSK602 with modified polylinker	Escolar *et al*. (2001)
pDMhpaA	pDSK604 derivative encoding HpaA-c-Myc	K. Hahn and U. Bonas (unpublished)
pDMhpaB	pDSK604 derivative encoding HpaB-c-Myc	Büttner *et al*. (2004)
pDMhpaC	pDSK604 derivative encoding HpaC-c-Myc	Büttner *et al*. (2006)
pDMhrcL	pDSK604 derivative encoding HrcL-c-Myc	Lorenz and Büttner (2009)
pDMhrpB2	pDSK602 derivative encoding HrpB2-c-Myc	Lorenz *et al*. (2008b)
pDMxopC	pDSK602 derivative encoding XopC-c-Myc	Büttner *et al*. (2007)
pDSF300	pDSK602 derivative encoding AvrBs3-FLAG	Van den Ackerveken *et al*. (1996)
pGEX-2TKM	GST expression vector; p*_tac_* GST *lacI*^q^ pBR322 *ori;* Ap^r^, derivative of pGEX-2TK with polylinker of pDSK604	Stratagene; Escolar *et al*. (2001)
pGhrcU	pGEX-2TKM derivative encoding GST–HrcU	Lorenz *et al*. (2008b)
pGhrcU_Y318D_	pGEX-2TKM derivative encoding GST–HrcU_Y318D_	This study
pGhrcU_N264A_	pGEX-2TKM derivative encoding GST–HrcU_N264A_	This study
pGhrcU_P265A_	pGEX-2TKM derivative encoding GST–HrcU_P265A_	This study
pGhrcU_P265G_	pGEX-2TKM derivative encoding GST–HrcU_P265G_	This study
pGhrcU_255–357_	pGEX-2TKM derivative encoding GST–HrcU_255–357_	Lorenz *et al*. (2008b)
pGhrcU_255–357/Y318D_	pGEX-2TKM derivative encoding GST–HrcU_255–357/Y318D_	This study
pGhrcU_255–357/N264A_	pGEX-2TKM derivative encoding GST–HrcU_255–357/N264A_	This study
pGhrcU_255–357/P265A_	pGEX-2TKM derivative encoding GST–HrcU_255–357/P265A_	This study
pGhrcU_255–357/P265G_	pGEX-2TKM derivative encoding GST–HrcU_255–357/P265G_	This study
pOK1	Suicide vector; *sacB sacQ mobRK2* oriR6K; Sm^r^	Huguet *et al*. (1998)
pOKΔhrcU	Derivative of pOK carrying the flanking regions of *hrcU*	This study
pOKΔhrcU_C_	Derivative of pOK carrying the flanking regions of *hrcU_265–357_*	This study
pOKhrcU_Y318D_	Derivative of pOK carrying *hrcU_Y318D_*	This study
pOKhrcU_P265G_	Derivative of pOK carrying *hrcU_P265G_*	This study
pRK2013	ColE1 replicon, TraRK^+^ Mob^+^; Km^r^	Figurski and Helinski (1979)
pUC119	ColE1 replicon; Ap^r^	Vieira and Messing (1987)

Ap, ampicillin; Km, kanamycin; Rif, rifampicin; Sm, spectinomycin; Gm, gentamicin; ^r^, resistant.

### Plant material and plant inoculations

The near-isogenic pepper cultivars Early Cal Wonder (ECW) and ECW-10R (Kousik and Ritchie, 1998; Astua-Monge *et al*., 2000) were grown and inoculated with *X. campestris* pv. *vesicatoria* as described previously (Bonas *et al*., 1991). Briefly, bacteria were inoculated into the intercellular spaces of leaves with a needle-less syringe at concentrations of 2 × 10^8^ colony-forming units (cfu) ml^−1^ in 1 mM MgCl_2_ if not stated otherwise. The appearance of disease symptoms and the HR were scored over a period of one to eleven dpi. For the better visualization of the HR, leaves were bleached in 70% ethanol. Experiments were repeated at least three times. For *in planta* growth curves, bacteria were inoculated at a density of 10^4^ cfu ml^−1^ into leaves of susceptible ECW plants. Bacterial counts were determined over a period of 7–10 dpi as described (Bonas *et al*., 1991).

### Generation of *X. campestris* pv. *vesicatoria hrcU* mutants

To create a 1047 bp in-frame deletion of *hrcU* (deletion of codons 3–351), we amplified the flanking regions of *hrcU* including the first 6 and the last 23 bp of the gene by PCR and cloned the PCR products into the ApaI/SalI sites of the suicide plasmid pOK1. The resulting construct pOKΔhrcU was conjugated into *X. campestris* pv. *vesicatoria* strains 85-10 and 85*. Double cross-overs resulted in *hrcU* deletion mutants that were selected as described previously (Huguet *et al*., 1998). Sequences of primers used in this study are available upon request.

For the generation of a *hrcU_265–357_* deletion mutant (deletion of codons 265–357), 750 bp of both flanking regions were amplified by PCR and cloned into the ApaI/SalI sites of pOK1. The resulting construct pOKΔhrcU_C_ was conjugated into strains 85-10 and 85*. Double cross-overs resulted in strains 85-10Δ*hrcU_265–357_* and 85*Δ*hrcU_265–357_* respectively.

For the introduction of the Y318D mutation into genomic *hrcU*, we amplified 800 bp fragments flanking codon 318 of *hrcU* with a 9 bp overlap that spans codons 317–319 of *hrcU*. Both amplicons contained a CTG to CTT exchange (silent mutation of codon 317) which creates a BclI site and a TAT to GAT exchange which leads to an exchange of Y318 by D318. PCR products were digested with XbaI/BclI and BclI/SalI, respectively, and cloned into the XbaI/SalI sites of plasmid pOK1. The resulting construct pOKhrcUY318D was conjugated into strain 85-10, 85-10Δ*hpaC*, 85* and 85*Δ*hpaC*. Double cross-overs resulted in strains 85-10*hrcU_Y318D_*, 85-10*hrcU_Y318D_*Δ*hpaC*, 85*hrcU_Y318D_ and 85*hrcU_Y318D_Δ*hpaC* respectively.

To introduce a point mutation into genomic *hrcU* leading to the P265G exchange, we amplified 750 bp fragments flanking codon 265 of *hrcU* with a 9 bp overlap that spans codons 265–267. Both amplicons contained a CCG to GGT exchange (mutation of codon 265) which creates a KpnI site and leads to the P265G mutation. PCR products were digested with XbaI/KpnI and KpnI/BamHI, respectively, and cloned into the XbaI/SalI sites of plasmid pOK1. The resulting construct pOKhrcU_P265G_ was conjugated into strains 85-10, 85* and 85*Δ*hpaC*. Double cross-overs resulted in strains 85-10*hrcU_P265G_*, 85*hrcU_P265G_ and 85*hrcU_P265G_Δ*hpaC* respectively.

### Generation of Golden Gate-expression constructs

For the generation of expression constructs encoding c-Myc epitope-tagged HrcU derivatives, *hrcU* or *hrcU* fragments encoding amino acids 265–357 and 206–357, respectively, were amplified by PCR and cloned into the Golden Gate-compatible expression vector pBRM in a one step restriction–ligation reaction as described (Engler *et al*., 2008). pBRM contains a single *lac* promoter and allows expression of genes in fusion with a C-terminal c-Myc epitope-encoding sequence (Szczesny *et al*., 2010). The Golden Gate system is based on type IIs restriction enzymes (e.g. BsaI) that cut DNA outside of the enzyme's recognition site. For the generation of *hrcU_Y318D_-c-myc* and *hrcU_P265G_-c-myc* expression constructs, *hrcU_Y318D_* and *hrcU_P265G_* were amplified by PCR from strains 85-10*hrcU_Y318D_* and 85-10*hrcU_P265G_*, respectively, and cloned into pBRM. Furthermore, *xopA*, *xopE2* and *xopJ* were amplified from *X. campestris* pv. *vesicatoria* strain 85-10 and cloned into pBRM. Expression constructs are listed in Table 1.

To generate HrcU point mutant derivatives with amino acid exchanges within the NPTH motif, codons 1–271 and 271–357 of *hrcU* were amplified by PCR. Both amplicons contained a 4 bp overlap and were cloned into pBRM in a single restriction–ligation reaction, generating pBRMhrcU. Individual mutations of codons 264–268, which replaced the amino acids N, P, T and H by A, respectively, were introduced by primer sequences (codon 264: AAC exchanged by GCC, codon 265: CCG exchanged by GCG, codon 266: ACC exchanged by GCC and codon 267: CAT exchanged by GCT). Similarly, for the introduction of point mutations into the NPTH motif of HrcU_206–357_-c-Myc, codons 206–271 and 271–357 of *hrcU*, respectively, were amplified by PCR and amplicons were cloned into pBRM as described above.

### Generation of pGEX constructs

To construct GST–HrcU_265–357_ and GST–HrcU_268–357_ fusion proteins, corresponding *hrcU* fragments were amplified by PCR and cloned into the EcoRI/XhoI sites of pGEX, downstream and in frame with the GST-encoding sequence. For the generation of GST–HrcU_Y318D_ and GST–HrcU_255–357/Y318D_ fusion proteins, *hrcU_Y318D_* and *hrcU_255–357/Y318D_* were amplified by PCR from strain 85-10*hrcU_Y318D_* and cloned into the EcoRI/XhoI sites of pGEX. To construct expression constructs encoding GST–HrcU_N264A_, GST–HrcU_P265A_, GST–HrcU_P265G_, GST–HrcU_255–357(N264A)_, GST–HrcU_255–357(P265A)_ and GST–HrcU_255–357(P265G)_, respectively, corresponding *hrcU* fragments were amplified from pBRMhrcU_N264A_, pBRMhrcU_P265A_ and pBRMhrcU_P265G_ and cloned into pGEX as described above.

### T3S assays and immunoblot analyses

Type III secretion assays were performed as described previously (Rossier *et al*., 1999). Briefly, bacteria were incubated in minimal medium A at pH 5.3 and equal amounts of bacterial total-cell extracts and culture supernatants were analysed by SDS-PAGE and immunoblotting (Rossier *et al*., 1999). In this study, we used polyclonal antibodies specific for HrpF (Büttner *et al*., 2002), XopA (Noël *et al*., 2002), AvrBs3 (Knoop *et al*., 1991) and HrpB2 (Rossier *et al*., 2000), respectively, and monoclonal anti-c-Myc (Roche Applied Science, Mannheim, Germany) and anti-GST antibodies (GE Healthcare, Munich, Germany). Horseradish peroxidase-labelled anti-rabbit, anti-mouse and anti-goat antibodies (GE Healthcare) were used as secondary antibodies. Antibody reactions were visualized by enhanced chemiluminescence (GE Healthcare). Experiments were repeated at least two times. Blots were routinely reacted with an antibody specific for the intracellular protein HrcN (Rossier *et al*., 2000) to ensure that no bacterial lysis had occurred (data not shown).

### GST pull-down assays

For GST pull-down assays, GST and GST fusion proteins were synthesized in *E. coli* BL21(DE3). Bacterial cells from 50 ml of cultures were resuspended in phosphate-buffered saline (PBS) and broken with a French press. Insoluble cell debris was removed by centrifugation and soluble GST and GST fusion proteins were immobilized on a glutathione sepharose matrix according to the manufacturer's instructions (GE Healthcare). Unbound proteins were removed by washing twice with PBS and the glutathione sepharose matrix was incubated with 600 µl of *E. coli* cell lysates containing c-Myc epitope-tagged derivatives of the putative interaction partners for 2 h at 4°C. Unbound proteins were removed by washing four times with PBS and bound proteins were eluted with 10 mM reduced glutathione at room temperature for 2 h. Ten microlitres of total protein lysates and 20 µl eluted proteins were analysed by SDS-PAGE and immunoblotting.
