# Supplementary material for: Secretion of early and late substrates of the type III secretion system from Xanthomonas is controlled by HpaC and the C-terminal domain of HrcU
Source: Mol Microbiol. 2011 Jan;79(2):447–67. doi: 10.1111/j.1365-2958.2010.07461.x (PMC3040844; doi:10.1111/j.1365-2958.2010.07461.x)
Supplement: Supplementary file 1 [file mmi0079-0447-SD1.pdf]

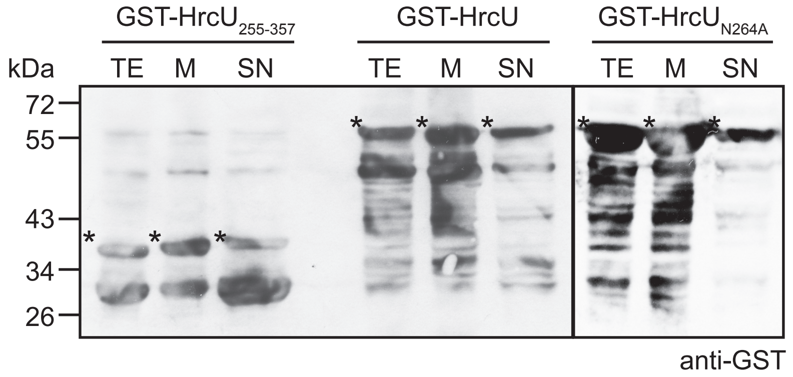

Lorenz and Büttner
Figure S1

Supplementary Fig. 1 GST-HrcU and derivatives are present in the soluble fractions of *E. coli* lysates.

E. coli cells containing GST-HrcU$_{255-357}$, GST-HrcU and GST-HrcU$_{N264A}$, respectively, were resuspended in PBS and lysed with a French Press. After centrifugation the pellet (P) containing insoluble proteins and cell debris was resuspended in 1 ml PBS. Total cell extracts (TE) and equal amounts of the resuspended pellet and the supernatant (SN) which contains the soluble proteins were analyzed by immunoblotting using a GST-specific antibody. Asterisks indicate full-length GST fusion proteins, additional signals correspond to degradation products.
